# Novel agents and regimens in acute myeloid leukemia: latest updates from 2022 ASH Annual Meeting

**DOI:** 10.1186/s13045-023-01411-x

**Published:** 2023-03-03

**Authors:** Katherine W. DiNardo, Thomas W. LeBlanc, Hui Chen

**Affiliations:** Duke University School of Medicine, Durham, NC 27708 USA

**Keywords:** Acute myeloid leukemia, AML, Investigational therapies, Clinical research

## Abstract

Developments in investigational agents and novel regimens in acute myeloid leukemia (AML) were reported in the 2022 American Society of Hematology (ASH) annual meeting. Encouraging efficacy data were presented from first-in-human studies of two investigational menin inhibitors, SNDX-5613 and KO-539, in relapsed and refractory (R/R) acute myeloid leukemia (AML) with KMT2A rearrangement or mutant NPM1, with overall response rates (ORR) of 53% (32/60) and 40% (8/20), respectively. The addition of the novel drug pivekimab sunirine, a first-in-class antibody–drug conjugate targeting CD123, to azacitidine and venetoclax in R/R AML resulted in an ORR of 45% (41/91), which rose to 53% in those who were venetoclax naïve. Additional novel triplet treatment combinations included the addition of magrolimab, an anti-CD47 antibody, to azacitidine and venetoclax, with an ORR of 81% (35/43) in newly diagnosed AML, including an ORR of 74% (20/27) in TP53 mutated AML. The addition of the FLT3 inhibitor gilteritinib to azacitidine/venetoclax was also featured, with an ORR of 100% (27/27) in newly diagnosed AML and an ORR of 70% (14/20) in R/R AML.

**To the editor**

Each year, exciting new developments and innovations in hematology are presented at the American Society of Hematology (ASH) meeting. In this article, we review some of the most impressive developments in investigational agents and novel regimens in acute myeloid leukemia (AML) from ASH 2022.

## Menin inhibitors in AML

Several presentations featured exciting activity from the novel class of menin inhibitors, which target the interaction of menin with the epigenetic regulators of KMT2A in AML. This inhibits leukemogenesis in AML cases featuring KMT2A rearrangement (KMT2Ar) or mutant NPM1 (mNPM1), two previously untargeted genetic alterations that represent 3–11% and 30% of newly diagnosed AML patients, respectively [1, 2]. Dr. Issa reported the results of the AUGMENT-101 trial, the first-in-human phase 1 trial of the menin inhibitor SNDX-5613 (revumenib) in patients with relapsed or refractory (R/R) AML. Only the 60 patients with KMT2Ar or mNPM1 were included in the efficacy analysis. In this heavily pretreated population (median four prior therapies, 46% prior transplant), the overall response rate (ORR, included CR/CRh/CRp/CRi/MLFS) was 53% (32/60) with a CRc rate (CR/CRh/CRp) of 38% (23/60), of which 78% were MRD (minimal residual disease) negative. Notable side effects included asymptomatic QTc prolongation and grade 2 or less differentiation syndrome in 16% (11/68) of patients [3].

An alternative menin inhibitor KO-539 (ziftomenib) was featured in Dr. Erba’s presentation of preliminary results from the KOMET-001 first-in-human Phase 1/2 trial of ziftomenib in adults with R/R AML. Preliminary efficacy data showed that in a similarly heavily pretreated population, the 600 mg dose had meaningful efficacy, with an ORR of 40% (8/20) and CRc in 35% (7/20) in mNPM1 AML, although outcomes were worse with KMT2Ar AML (ORR 16.7%, CRc 11%). Differentiation syndrome (DS) was a notable adverse event, with at least grade 3 severity occurring in 27% (8/29) of KMT2Ar patients including 1 death, although no grade 3 DS occurred in mNPM1 patients. However, DS was associated with improved response (75% of mNPM1 patients), and DS severity decreased with improved protocol guidance [4].

## Novel regimens in AML

Pivekimab sunirine (PVEK, IMGN632) is a first-in-class antibody–drug conjugate (ADC) targeting CD123, which is expressed on most AML blasts, with a novel indolinobenzodiazepine pseudodimer (IGN) payload that alkylates DNA. In this phase 1b/2 study of the triplet regimen of PVEK, azacitidine (AZA), and venetoclax (VEN) presented by Dr. Daver, there was an ORR of 45% (41/91) with CRc of 25% (23/91), with higher responses (ORR 53% and CRc 38%) in the 47 patients who were VEN-naïve. The drug was well tolerated, with infusion reactions occurring in 22% (20/91) of patients, and only 5% discontinued treatment due to PVEK-related adverse events [5].

Dr. Daver also presented data from the phase 1/2 study of the novel triplet regimen of magrolimab, an anti-CD47 antibody that blocks the “don’t eat me” signal on leukemia cells, with AZA and VEN in older/unfit or high risk AML. The ORR in newly diagnosed AML was similar to prior reported responses with AZA/VEN, with ORR 81% (35/43) including an ORR of 74% (20/27) with TP53 mutations. Median OS (mOS) was not reached in newly diagnosed non-secondary AML patients, with mOS 7.6 months in untreated secondary AML with TP53 mutation. Responses in R/R AML were more modest, with prior VEN exposed patients faring poorly (ORR 11%), resulting in closure of this study arm. Grade 3 anemia occurred in 23% (18/79), and median hemoglobin drop was 1.2 g/dL after magrolimab infusion [6].

Another exciting novel triplet regimen was the addition of gilteritinib, an oral FLT3 inhibitor, to AZA/VEN in FLT3-mutated AML. The ORR in this phase I/II study presented by Dr. Short was 100% (27/27) with 92% CR in newly diagnosed patients, with median OS not yet reached and 1-year OS 85%. The ORR was 70% (14/20) in R/R patients, with 20% (4/20) achieving CR, and a median OS of 5.8 months. Outcomes were better in those who had not received prior VEN or gilteritinib, with mOS 10.5 months [7].

Overall, the ASH 2022 annual meeting showcased several notable advances in the field of investigational therapies in AML, as summarized in Tables 1 and 2, including encouraging efficacy of the novel menin inhibitors SNDX-5613 and KO-5319, the novel ADC PVEK, and the novel additions of magrolimab and gilteritinib to AZA/VEN.

**Table 1:** Properties of featured novel agents and treatment combinations in AML from ASH 2022

Drug	Regimen Backbone	Indication	Mechanism	References
Magrolimab	AZA/VEN	ND AML	Anti-CD47 antibody	[6]
Gilteritinib	AZA/VEN	ND and R/R AML	FLT3 inhibitor	[7]
SNDX-5613 (Revumenib)	N/A	R/R AML	Menin inhibitor	[3]
KO-539 (Ziftomenib)	N/A	R/R AML	Menin inhibitor	[4]
Pivekimab sunirine (PVEK)	AZA/VEN	R/R AML	Antibody–drug conjugate targeting CD123	[5]

*AZA* azacitidine, *VEN* venetoclax, *ND* newly diagnosed, *R/R* relapsed/refractory

**Table 2:** Outcomes of clinical trials of featured novel agents and treatment combinations in AML from ASH 2022

Drug	Indication	ORR	CRc	Notable side effects	References
Magrolimab	ND AML	81%	72%	Anemia, infusion reactions	[6]
Gilteritinib	ND and R/R AML	100% (ND) 70% (R/R)	92% (ND) 20% (R/R)	Infection	[7]
SNDX-5613 (Revumenib)	R/R AML	53%	38%	Differentiation syndrome	[3]
KO-539 (Ziftomenib)	R/R AML	40%	35%	Differentiation syndrome, pneumonitis	[4]
Pivekimab sunirine (PVEK)	R/R AML	45%	25%	Infusion reactions	[5]

*ND* newly diagnosed, *R/R* relapsed/refractory, *ORR* overall response rate, encompassing CR/CRh/CRp/CRi/MLFS, *CRc* composite complete remission, encompassing CR/CRh/CRp

## Data Availability

The material supporting the conclusion of this study has been included within the article.

## Abbreviations

ADC: Antibody-drug conjugate
AML: Acute myeloid leukemia
ASH: American Society of Hematology
AZA: Azacitidine
CR: Complete remission (blasts < 5% by morphology, ANC > 1,000, platelets > 100,000)
CRc: Composite complete remission
CRh: Complete remission with partial hematologic recovery (CR but ANC 500–1,000 and platelets 50,000–100,000)
CRi: Complete remission with incomplete hematologic recovery (CR but ANC 500–1,000 or platelets 50,000–100,000)
CRp: Complete remission with incomplete platelet recovery (CR but platelet count does not meet criteria)
DS: Differentiation syndrome
KMT2A: Histone-lysine-N-methyltransferase 2A
MLFS: Morphologic leukemia-free state (CR but ANC < 1,000 and platelets < 100,000)
MRD: Minimal residual disease
N: Number of patients
ND: Newly diagnosed
NPM1: Nucleophosmin 1
ORR: Overall response rate
OS: Overall survival
PVEK: Pivekimab sunirine
R/R: Relapsed/refractory
VEN: Venetoclax

## References

[1] Falini B, Mecucci C, Tiacci E, Alcalay M, Rosati R, Pasqualucci L (2005). Cytoplasmic nucleophosmin in acute myelogenous leukemia with a normal karyotype. N Engl J Med.

[2] Basecke J, Whelan JT, Griesinger F, Bertrand FE (2006). The MLL partial tandem duplication in acute myeloid leukaemia. Br J Haematol.

[3] Issa GC, Aldoss I, DiPersio JF, Cuglievan B, Stone RM, Arellano ML (2022). The Menin Inhibitor SNDX-5613 (revumenib) Leads to Durable Responses in Patients (Pts) with KMT2A-Rearranged or NPM1 Mutant AML: Updated Results of a Phase (Ph) 1 Study. Blood.

[4] Erba HP, Fathi AT, Issa GC, Altman JK, Montesinos P, Patnaik MM, et al. Update on a Phase 1/2 First-in-Human Study of the Menin-KMT2A (MLL) Inhibitor Ziftomenib (KO-539) in Patients with Relapsed or Refractory Acute Myeloid Leukemia. Oral session presented at: 64th Annual Meeting of the American Society of Hematology; 2022 Dec 10–13; New Orleans.

[5] Daver N, Montesinos P, Aribi A, Marconi G, Altman JK, Wang ES, et al. Broad Activity for the Pivekimab Sunirine (PVEK, IMGN632), Azacitidine, and Venetoclax Triplet in High-Risk Patients with Relapsed/Refractory Acute Myeloid Leukemia (AML). Oral session presented at: 64th Annual Meeting of the American Society of Hematology; 2022 Dec 10–13; New Orleans.

[6] Daver N, Senapati J, Maiti A, Loghavi S, Kadia TM, DiNardo CD, et al. Phase I/II Study of Azacitidine (AZA) with Venetoclax (VEN) and Magrolimab (Magro) in Patients (pts) with Newly Diagnosed (ND) Older/Unfit or High-Risk Acute Myeloid Leukemia (AML) and Relapsed/Refractory (R/R) AML. Oral session presented at: 64th Annual Meeting of the American Society of Hematology; 2022 Dec 10–13; New Orleans.

[7] Short N, DiNardo CD, Daver N, Macaron W, Yilmaz M, Borthakur G, et al. Updated Results from a Phase I/II Study of the Triplet Combination of Azacitidine, Venetoclax and Gilteritinib for Patients with FLT3-Mutated Acute Myeloid Leukemi**a.** Oral session presented at: 64th Annual Meeting of the American Society of Hematology; 2022 Dec 10–13; New Orleans.

